# Probabilistic Shaping for Finite Blocklengths: Distribution Matching and Sphere Shaping

**DOI:** 10.3390/e22050581

**Published:** 2020-05-21

**Authors:** Yunus Can Gültekin, Tobias Fehenberger, Alex Alvarado, Frans M. J. Willems

**Affiliations:** 1 Signal Processing Systems Group, Information and Communication Theory Lab, Department of Electrical Engineering, Eindhoven University of Technology, 5600 MB Eindhoven, The Netherlands; a.alvarado@tue.nl (A.A.); f.m.j.willems@tue.nl (F.M.J.W.); 2 ADVA Optical Networking, 82152 Munich, Germany; tfehenberger@adva.com

**Keywords:** shaping gap, constellation shaping, probabilistic amplitude shaping, distribution matching, sphere shaping

## Abstract

In this paper, we provide a systematic comparison of distribution matching (DM) and sphere shaping (SpSh) algorithms for short blocklength probabilistic amplitude shaping. For asymptotically large blocklengths, constant composition distribution matching (CCDM) is known to generate the target capacity-achieving distribution. However, as the blocklength decreases, the resulting rate loss diminishes the efficiency of CCDM. We claim that for such short blocklengths over the additive white Gaussian noise (AWGN) channel, the objective of shaping should be reformulated as obtaining the most energy-efficient signal space for a given rate (rather than matching distributions). In light of this interpretation, multiset-partition DM (MPDM) and SpSh are reviewed as energy-efficient shaping techniques. Numerical results show that both have smaller rate losses than CCDM. SpSh—whose sole objective is to maximize the energy efficiency—is shown to have the minimum rate loss amongst all, which is particularly apparent for ultra short blocklengths. We provide simulation results of the end-to-end decoding performance showing that up to 1 dB improvement in power efficiency over uniform signaling can be obtained with MPDM and SpSh at blocklengths around 200. Finally, we present a discussion on the complexity of these algorithms from the perspectives of latency, storage and computations.

## 1. Introduction

Coded modulation (CM), which combines multi-level modulation with forward error correction (FEC), is indispensable for digital communication strategies targeting high transmission rates. To realize CM, different techniques have been proposed in the literature, such as multilevel coding (MLC) [1,2], trellis CM [3], and bit-interleaved CM (BICM) [4,5,6,7,8]. Among the many proposed CM architectures, the de-facto standard is to combine a high-order modulation format with a binary FEC code using a binary labeling strategy, frequently in the absence of an interleaver, and to use bit-metric decoding (BMD) at the receiver [7], which corresponds to the BICM paradigm.

As the modulation order increases, the maximum rate that can be achieved with uniform signaling starts to suffer from a loss with respect to the capacity of the additive white Gaussian noise (AWGN) channel. As an example, the maximum achievable information rate (AIR) for MLC in combination with multi-stage decoding (MSD) [2] is the mutual information (MI) of the channel input and output. If a uniform signaling strategy is employed with MLC-MSD, the MI is bounded away from capacity. This gap is called the *shaping gap* and is up to 0.255 bits per real channel use (bit/1-D) for the AWGN channel. When translated into an increase in required signal-to-noise ratio (SNR) to obtain a certain MI, this so-called *ultimate shaping gap* corresponds to a 1.53 dB loss in power efficiency [9].

**Figure 1:**
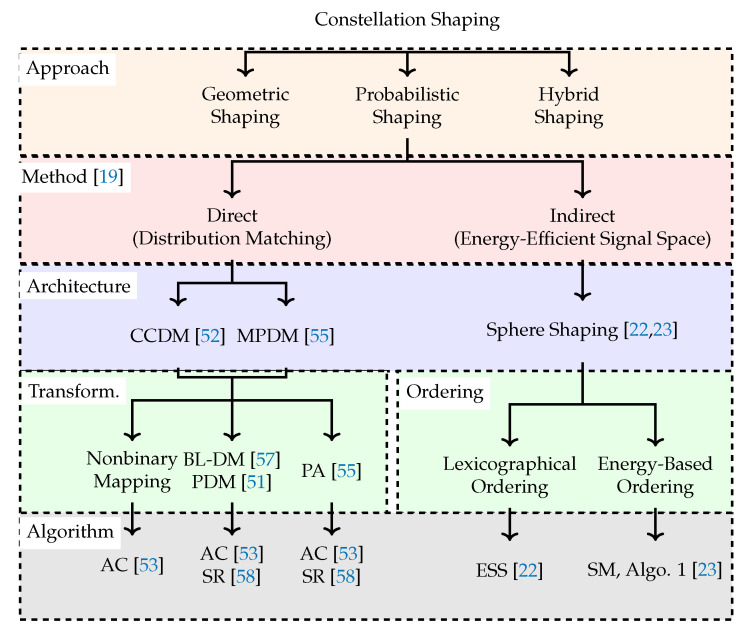
Taxonomy of shaping in the context of probabilistic amplitude shaping (PAS). We focus on the schemes that are evaluated in this work.

There exist numerous techniques in the literature, most of them proposed in the late 1980s and early 1990s, that attempt to close the shaping gap. Motivated by the fact that the capacity-achieving distribution for the AWGN channel is Gaussian, these techniques fundamentally take one of the following approaches. The first is to construct a signal constellation with a Gaussian-like geometry as illustrated in (Figure 4.49 in [10]), which is called geometric shaping (GS) [11,12,13,14,15,16,17,18]. The other approach is to induce a Gaussian-like distribution over the signal structure, which is called probabilistic shaping (PS) [19,20,21,22,23]. PS techniques can be further classified into two subgroups using the terminology introduced by Calderbank and Ozarow in [19]. The *direct* approach is to start with a target distribution (which is typically close to the capacity-achieving distribution) on a low-dimensional signal structure and have an algorithm try to obtain it [19,21]. Following recent literature [24], the direct approach can also be called distribution matching (DM). The *indirect* approach is to start with a target rate and bound the *n*-dimensional signal structure by a sphere, which we call sphere shaping (SpSh) [22,23]. Here, a (sampled) Gaussian distribution is obtained indirectly (when n→∞) as a by-product. Finally, there exist some *hybrid shaping* approaches in which GS and PS are combined [25,26,27]. We refer to (Section 4.5 in [10]) for a detailed discussion on GS, and to (Chapter 4 in [10]) and (Section II in [28]) on PS. GS, PS, and hybrid shaping are shown on the top layer of Figure 1 where the taxonomy of constellation shaping (as discussed in the current paper) is illustrated. We call this first layer *shaping approach*. On the second layer which we call *shaping method*, PS is split into two following the Calderbank/Ozarow terminology [19].

In the context of BICM, constellation shaping techniques again attracted a considerable amount of attention in the 2000s. GS was investigated for BICM in [29,30,31], and PS was studied in [32,33,34,35]. An iterative demapping and decoding architecture with PS was proposed in [36]. The achievability of the so-called generalized MI (GMI) was shown for independent but arbitrarily distributed bit-levels in [37]. In [38], it was demonstrated that the GMI is a nonconvex function of the input bit distribution, i.e., the problem of computing the input distribution that maximizes GMI is nonconvex. An efficient numerical algorithm to compute optimal input distributions for BICM was introduced in [39]. The effect of mismatched shaping, i.e., not using the true symbol probabilities or reference constellation at the receiver, was examined in [40]. The achievable rates, error exponents and error probability of BICM with PS were analyzed in [41]. Constellation shaping was investigated for BICM at low SNR in [42]. PS in BICM was considered for Rayleigh fading channels in [43,44].

**Figure 2:**
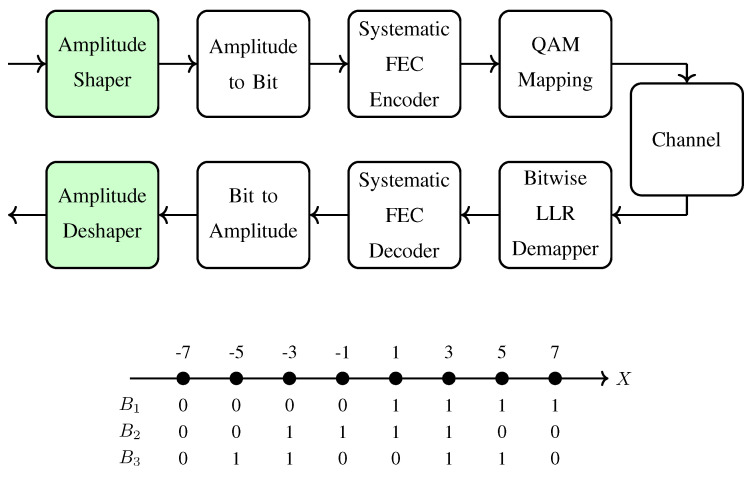
(**Top**) Block diagram of the PAS architecture. Amplitude shaping blocks (green boxes) are examined in the current paper. (**Bottom**) The binary reflected Gray code (BRGC) for 8-ary amplitude- shift keying (8-ASK). A quadrature amplitude modulation (QAM) symbol is the concatenation of two ASK symbols.

Recently, probabilistic amplitude shaping (PAS) has been proposed to provide low-complexity integration of shaping into existing binary FEC systems with BMD [28]. PAS uses a reverse concatenation strategy where the shaping operation precedes FEC coding, as shown in Figure 2 (top). This construction has been first examined for constrained coding problems [45]. A corresponding soft-decision decoding approach for this structure was studied in [46]. PAS can be considered as an instance of the Bliss architecture [45] where in the outer layer a shaping code is used, and then in the inner layer parity symbols are added. The main advantage of this structure is that amplitude shaping can be added to existing CM systems as an outer code. (We note here that in optical communication literature, the term “intensity modulation” is used to designate amplitude modulation in general. To be consistent with the constellation shaping literature, we prefer to use the term “amplitude shaping” in this study.) In addition to closing the shaping gap, PAS also has rate adaptation functionality in the shaping layer. This means that instead of using many FEC codes of different rates to obtain a granular set of transmission rates, the rate can be adjusted by the amplitude shaper with a fixed FEC code. Owing to these advantages, PAS has attracted a lot of attention. PAS has been combined with low-density parity-check (LDPC) codes [28], polar codes [47] and convolutional codes [48]. Its performance has been evaluated over the AWGN channel [28], optical channels [49,50], wireless channels [48] and parallel channels with channel state information available at the transmitter [51].

The key building blocks of the PAS framework are the amplitude shaper and deshaper, i.e., the green boxes in Figure 2 (top). The function of the amplitude shaper is to map uniform binary sequences to shaped amplitude sequences in an invertible manner. A careful selection of the set of sequences that can be outputted by the shaper with the aim of matching a target distribution (direct approach) or constructing an energy-efficient signal space (indirect approach) results in improvement in overall performance. We call the way this selection is accomplished *shaping architecture* which affects the performance of PAS. (For channels other than the AWGN channel, metrics other than energy may be used to assess the efficiency of the signal space. In such cases, boundary structures other than spheres may lead to more efficient signal spaces. Equivalently, capacity-achieving distribution is expected to be non-Gaussian for these channels.) On the other hand, the actual implementation of this architecture is called here *shaping algorithm* and determines the complexity of attaining this performance. The third and fifth layers of Figure 1 illustrate shaping architectures and algorithms, respectively. The difference between the shaping architecture and the underlying algorithm is discussed in detail in Section 2.4.

For the initial proposal of PAS [28], constant composition distribution matching (CCDM) was employed as the shaping architecture [52]. The basic principle of CCDM is to utilize amplitude sequences having a fixed empirical distribution that is information-theoretically close to the target distribution. To this end, a constant composition constraint is put on the output sequences such that all have the same amplitude composition. To realize such a mapping, arithmetic coding (AC) is used in a way similar to [53]. Although CCDM has vanishing rate loss for asymptotically large blocklengths [52], it has two fundamental drawbacks. First, as recently shown in [54] and (Figure 4 in [55]), CCDM suffers from high rate losses as the blocklength decreases. Second, CCDM is implemented based on AC which requires sequential processing [53] and (Chapter 5 in [56]).

To replace CCDM in the short-to-moderate blocklength regime and to provide more hardware-friendly implementations, improved techniques have been devised. The most prominent DM examples other than CCDM include multiset-partition DM (MPDM) [55] and product DM (PDM) [51,57]. Briefly stated, MPDM uses different compositions and expands the set of output sequences to achieve smaller rate losses than CCDM. With the same objective, PDM internally uses multiple binary matchers to generate the desired distribution as a product distribution (A symbol-level product distribution can be written as the product of bit-level distributions (Equation (14) in [51]). In the context of BICM, product distributions were studied extensively in [41]). In [58], a parallel-amplitude (PA) architecture is proposed for DM to enable even higher degrees of parallelization. Also in [58], subset ranking (SR) is introduced as an alternative to the conventional AC method for binary-output CCDM. As for direct shaping methods, enumerative sphere shaping (ESS) and shell mapping (SM) are notable SpSh algorithms which are initially proposed in [22,59] respectively. ESS is recently considered in PAS framework [48,60,61,62,63], as well as SM in [64]. Furthermore, low-complexity implementation ideas for both of these algorithms have been presented in [65].

The fourth layer in Figure 1 which we call *transformation* for DM and *ordering* for SpSh designates the way a shaping algorithm formulates a solution to the problem defined by the shaping architecture. As an example, CCDM considers sequences having the same composition [52]. By realizing a binary-to-nonbinary transformation with AC [52,53], CCDM can directly be used to produce amplitude sequences. On the other hand, separate binary-to-binary transformations can be employed for different bit-levels using AC [53] or SR [58]. Then these bit-levels can be combined such that the corresponding channel input distribution is close to the capacity-achieving distribution [51,57]. As another example, SpSh considers amplitude sequences inside a sphere. ESS orders these sequences lexicographically [22], while SM and (Algorithm 1 in [23]) order them based on their energy.

Other shaping schemes have been proposed that are briefly listed in the following. A detailed analysis of them is outside the scope of this manuscript. The concept of a “mark ratio controller” was proposed for low-complexity implementation of BL-DM in [66,67]. In the streaming DM of [68] and the prefix-free code distribution matching with framing of [69,70], switching is performed between two (or more) variable-length shaping codes such that the output is always of fixed length. In [71], a “multi-composition” idea similar to [55] was applied to BL-DM. The authors of [72] provided a finite-precision implementation for AC-CCDM. In [73], a shaper based on ESS was introduced to shape a subset of the amplitude bit-levels, which is referred to as partial ESS. The authors of [74] introduced the “hierarchical” DM which realizes a nonuniform distribution with hierarchical lookup tables (LUTs) [75]. An approximate sphere shaping implementation based on Huffman codes was proposed in [76].

In this work, we examine DM and SpSh methods. The contributions of this paper are threefold. First, we provide a systematic comparison of several PS architectures for PAS framework. Second, using rate loss as well as information rates for finite-length shapers as the performance metrics, we claim that shaping strategies which aim to construct energy-efficient signal sets are more effective than the techniques which focus on matching distributions for the AWGN channel. For the analyzed schemes, this means that MPDM and SpSh, are more efficient for short blocklengths than CCDM whose sole objective is to obtain the capacity-achieving distribution. Our claim is then verified via frame error rates (FERs) that are obtained in end-to-end decoding simulations of the PAS system employing long and short systematic LDPC codes from [77,78] respectively. The improvements in power efficiency that we obtained during end-to-end decoding simulations are consistent with the predictions made by finite-length information rates. The third contribution of this paper is to provide a discussion on the required storage, computational complexity, and latency of different DM and SpSh algorithms.

The paper is organized as follows. The first part is tutorial-like. In Section 2, background information on amplitude shaping is provided. In Section 3, uniform and shaped signaling schemes are described. Section 4 reviews DM and SpSh schemes from shaping architecture and algorithmic implementation perspectives. The second part of the paper is reserved for the comparison of four amplitude shaping architectures. Rate losses, information rates, and end-to-end decoding performance of PAS are studied in Section 5. Section 6 is devoted to a high-level discussion on latency and complexity of the schemes under consideration. Finally, conclusions are given in Section 7.

## 2. Preliminaries

### 2.1. Notation and Definitions

We use capital letters *X* to denote random variables, lower case letters *x* to specify their realizations. Random vectors of length *n* are indicated by $X^{n}$ while their realizations are denoted by $x^{n}$. Element-wise multiplication of $x^{n}$ and $y^{n}$ is shown by $x^{n}y^{n}$. Calligraphic letters X represent sets. We use XY to denote {xy:x∈X,y∈Y}. The *n*-fold Cartesian product of X with itself is denoted by $X^{n}$. Boldface capital letters P specify matrices. Probability functions (density or mass) over X are denoted by $P_{X}\left(x\right)$. The probability density function of *Y* conditioned on *X* is indicated by $P_{Y|X}\left(y|x\right)$.

The discrete-time AWGN channel output is given at time i=1,2,⋯,n by $Y_{i}=X_{i}+Z_{i}$, where $Z_{i}$ is the noise which is independent of the input $X_{i}$, and drawn from a zero-mean Gaussian distribution with variance $\sigma^{2}$. The noise $Z_{i}$ is also independent over time *i*. There is an average power constraint $E[X^{2}]\leq P$, where E[·] is the expectation operator. The SNR is $E\left[X^{2}\right]/\sigma^{2}$.

The capacity of the AWGN channel is given by

(1) $$\begin{array}{c} C=\frac{1}{2}\log_{2}\left(1+\mathrm{SNR}\right), \end{array}$$

in bit/1-D. This capacity can be achieved as n→∞ by employing a codebook (set of input sequences) in which all the codewords (input sequences) are generated with entries independent and identically distributed according to a zero-mean Gaussian with variance *P* (Chapter 9 in [79]). The corresponding random coding argument shows that channel input sequences, drawn from a Gaussian distribution, are likely to lie inside an *n*-dimensional ball of squared radius nP(1+ε) for any ε>0, when n→∞. This motivates to select the signal points from within an *n*-ball, or equivalently, to use an *n*-sphere as the signal space boundary, in order to achieve capacity. For a more detailed discussion on the asymptotic duality of Gaussian distributions and *n*-spherical signal spaces for large *n*, we refer the reader to, e.g., (Section IV-B in [9]).

### 2.2. Discrete Constellations and Amplitude Shaping

We consider $2^{m}$-ary amplitude-shift keying (ASK) alphabets $X=\{\pm 1,\pm 3,\cdots ,\pm \left(2^{m}-1\right)\}$, which can be factorized as X=SA. Here S={±1} and $A=\{1,3,\cdots ,2^{m}-1\}$ are the sign and amplitude alphabets, respectively. The cardinality of the amplitude alphabet is $n_{a}=\left|A\right|$. Motivated by the fact that the capacity-achieving distribution for the AWGN channel is symmetric around zero, we restrict our attention to the amplitude distribution $P_{A}\left(a\right)$, and assume that the sign distribution $P_{S}\left(s\right)$ is uniform and independent of the amplitudes. The distribution of the channel input X=SA is then $P_{X}\left(x\right)=P_{S}\left(s\right)P_{A}\left(a\right)$.

The distribution that maximizes the MI for ASK constellations subject to an average power constraint does not have a known analytical form. Instead, Maxwell-Boltzmann (MB) distributions

(2) $$P_{A}\left(a\right)=\left\{\begin{array}{ccc} K\left(\lambda \right)e^{-\lambda a^{2}} & , & \mathrm{for}a\in A,\\ 0 & , & \mathrm{otherwise}, \end{array}\right.$$

are used for shaping amplitudes, e.g., in [21,28]. As shown in (Table 5.1 in [80]), the difference in MI for the MB distribution and the capacity-achieving distribution is insignificant for ASK constellations. In (2), λ determines the variance of the distribution while K(λ) normalizes it.

Similarly, SpSh is also employed for amplitude shaping in the discrete domain [22,23]. In [54], it is shown that when an *n*-spherical region of $X^{n}$ is used as the signal space, the average distribution over A approaches an MB distribution as n→∞. The authors of [64] showed that at finite *n*, SpSh minimizes the informational divergence between the average distribution and an MB distribution.

To employ high-order modulation formats such as $2^{m}$-ASK for m≥2, a binary labeling strategy is necessary. A discussion on binary labeling can be found in (Section 2 in [8]). We assume that the binary label $B_{1}B_{2}\cdots B_{m}$ of a channel input *X* can be decomposed into a sign bit $B_{1}$ and amplitude bits $B_{2}B_{3}\cdots B_{m}$. In this paper, we assume that binary reflected Gray codes (BRGCs) are used for labeling (Defn. 2.10 in [8]).

**Example** **1** (Binary labeling)**.** *The BRGC is tabulated for 8-ASK in Figure 2 (bottom). Here, $B_{1}$ is symmetric around zero. Furthermore, when X has a distribution which is symmetric around zero, $B_{1}$ is uniform and stochastically independent of $B_{2}$ and $B_{3}$.*

### 2.3. Fundamentals of Amplitude Shaping Schemes

The amplitude shaper is a block that maps *k*-bit sequences to *n*-amplitude sequences in an invertible manner. The tasks of this block are (i) to create a *shaping codebook* $A^{⋆}\subseteq A^{n}$, and (ii) to realize a *shaping encoder* to index these sequences. The former task is related to the properties of the desired set $A^{⋆}$ while the latter deals with the algorithmic implementation of the mapping. This difference is discussed in detail in Section 2.4. In the remainder of this section, we introduce the concepts and parameters that are associated with the shaping techniques that will be investigated in this paper.

The energy of a sequence $a^{n}=\left(a_{1},a_{2},\cdots ,a_{n}\right)$ is given by

(3) $$\begin{array}{c} e\left(a^{n}\right)=\sum_{j=1}^{n}a_{j}^{2}. \end{array}$$

When *n*-sequences are represented as points in an *n*-dimensional (*n*-D) space, the set

(4) $$\begin{array}{c} A^{•}=\left\{a^{n}:e\left(a^{n}\right)\leq E^{•}\right\} \end{array}$$

consists of all amplitude sequences located inside or on the surface of the *n*-sphere of squared radius $E^{•}$. The zero-energy point is at the center of this sphere.

The composition of a sequence $a^{n}\in A^{n}$ is defined as $C=[n_{1},n_{2},\cdots ,n_{\left|A\right|}]$, where $n_{j}$ denotes the number of times the *j*th element of A occurs in $a^{n}$. The number of *n*-sequences with the same composition *C* is given by the multinomial coefficient

(5) $$\begin{array}{c} \mathrm{MC}\left(C\right)=\frac{n!}{n_{1}!n_{2}!\cdots n_{\left|A\right|}!}. \end{array}$$

For a set $A^{⋆}$ of amplitude sequences with average amplitude distribution $P_{A}\left(a\right)$ over A, the average energy per symbol is given by

(6) $$\begin{array}{c} E=\sum_{a\in A}P_{A}\left(a\right)a^{2}. \end{array}$$

The shaping rate of the set $A^{⋆}$ is defined as

(7) $$\begin{array}{c} R_{s}=\frac{\log_{2}\left|A^{⋆}\right|}{n} \end{array}$$

in bit/1-D. The input blocklength of a shaping algorithm that indexes sequences from the shaping set $A^{⋆}$ is

(8) $$\begin{array}{c} k=\left\lfloor \log_{2}\left(\left|A^{⋆}\right|\right)\right\rfloor \end{array}$$

in bits. It can be shown that the parameters of a shaping code $A^{⋆}$ satisfy the following inequality

(9) $$\begin{array}{c} H\left(A\right)\overset{\left(a\right)}{\geq }\frac{\log\left|A^{⋆}\right|}{n}\overset{\left(b\right)}{\geq }\frac{k}{n} \end{array}$$

where (a) is due to the finite blocklength *n* and (b) is due to the binary-input nature of the shaping algorithm, i.e., the rounding in (8). Here H(A) is the entropy of $P_{A}$ in bits. In (9), both (a) and (b) are satisfied with equality when n→∞ for asymptotically optimum amplitude shaping architectures. The rate loss of a shaping set $A^{⋆}$ with average distribution $P_{A}\left(a\right)$ can then be defined in bit/1-D as

(10) $$\begin{array}{c} R_{\mathrm{loss}}=H\left(A\right)-\frac{k}{n}. \end{array}$$

### 2.4. Shaping Architecture vs. Shaping Algorithm

The aforementioned shaping schemes have in common that they are aiming at solving an indexing problem, which is that the binary input of the shaper determines an amplitude sequence. At the receiver side, the inverse operation is carried out. For proper characterization and categorization of this indexing problem, it is insightful to differentiate between architectures and algorithms.

When we speak of the architecture, we mean the underlying principle behind the mapping operation, which in turn can be realized with various different algorithms as shown in the fifth layer of Figure 1. For instance, the principle of the CCDM architecture is that the sequences at the shaper output have a fixed number of occurrences of each amplitude, i.e., they have a fixed composition. Furthermore, the mapping algorithm can operate on one nonbinary or several binary subsets of the output sequence. Bit-level [51,57] and parallel-amplitude [58] designs are modifications to the conventional CCDM architecture that carry out such a transformation from one nonbinary to several binary DMs. Among all algorithms, a LUT is probably the simplest way to solve the CCDM indexing problem, yet the LUT size table is prohibitively large as it reaches gigabit size already for short blocklengths [76]. The original mapping method for a nonbinary-alphabet CCDM is AC (Section IV in [52]) which is modified from [53]. For binary-output CCDM, SR has recently been proposed as a low-serialism alternative to CCDM. MPDM [55] extends the CCDM principle (and thus architecture) by using variable-composition DM, yet internally uses CCDM methods for mapping and demapping.

As another example, the principle of SpSh architecture is that the sequences at the output of the shaper satisfy a maximum-energy constraint, i.e., they satisfy (4). The problem of indexing these sequences can be solved again by a using a LUT. On the other hand, ESS [22], SM [23] and (Algorithm 1 in [23]) are constructive algorithms to index sequences inside a sphere. The required storage and computational complexity of these algorithms are compared in Section 6. For further discussion on SM, we refer the reader to [23,81], (Chapter 8 in [82]) and (Section 4.3 in [10]).

## 3. Signaling Schemes

### 3.1. Uniform Signaling

**Figure 3:**
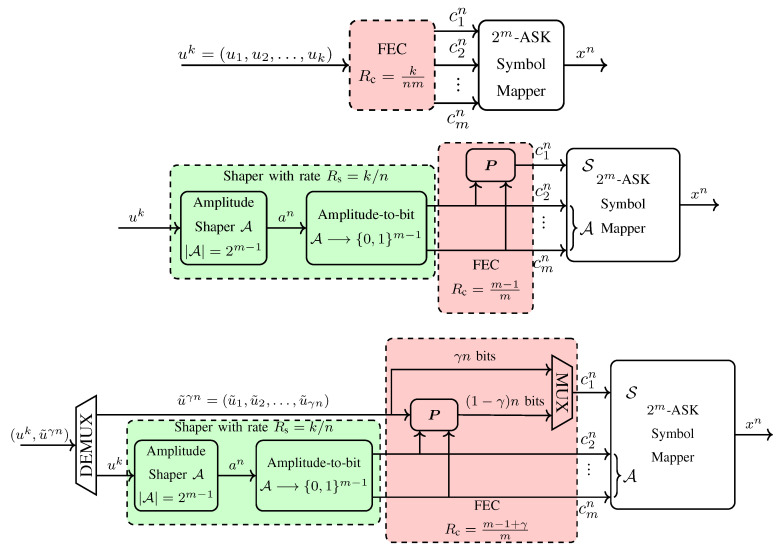
Signaling options: (**top**) uniform signaling with rate R=k/n bit/1-D, (**middle**) PAS with rate R=k/n bit/1-D (all information is on amplitudes), (**bottom**) modified PAS with rate R=k/n+γ bit/1-D (extra data is carried on signs).

In uniform signaling, a *k*-bit uniform sequence $u^{k}=\left(u_{1},u_{2},\cdots ,u_{k}\right)$ is encoded by a rate $R_{c}=k/n_{c}$ FEC code, as shown in Figure 3 (top). Afterwards, the coded sequence $c^{n_{c}}$ is divided into *m*-bit vectors $\left(c_{1},c_{2},\cdots ,c_{m}\right)$, each of which is mapped to a channel input symbol x∈X via the symbol mapper. Finally, assuming that $n_{c}/m=n$, the sequence $x^{n}\in X^{n}$ is transmitted over the channel. The transmission rate of this construction is R=k/n bit/1-D. We will compare the uniform and shaped signaling techniques at the same transmission rate *R*, as it is obviously the only fair comparison as recently discussed in (Section IV-A in [61]) and [83].

### 3.2. Probabilistic Amplitude Shaping

Böcherer et al. introduced in [28] the PAS framework which couples an outer shaping code and an inner FEC code to realize *shaped-coded modulation*. Figure 3 (middle) shows the basic PAS architecture where first, an amplitude shaping block maps a *k*-bit uniform information sequence $u^{k}$ to an *n*-amplitude sequence $a^{n}=\left(a_{1},a_{2},\cdots ,a_{n}\right)$ in an invertible manner, where $a_{j}\in A$ for j=1,2,⋯,n. After this mapping block, these amplitudes are transformed into bits using the last m−1 bits of the corresponding BRGC, i.e., the amplitude bits. We note that due to the shaped nature of $a^{n}$, the bits at the output of the amplitude-to-bit conversion in Figure 3 (middle) are nonuniform. These n(m−1) nonuniform bits $c_{2}^{n},c_{3}^{n},\cdots ,c_{m}^{n}$ are then used as the input of a systematic, rate $R_{c}=\left(m-1\right)/m$ FEC code which is specified by an *n*-by-nm parity-check matrix P. The *n*-bit parity output of this code is employed as the sign bit-level, i.e., the first bit of the BRGC, to determine the sign sequence $s^{n}=\left(s_{1},s_{2},\cdots ,s_{n}\right)$. Finally, $x^{n}=s^{n}a^{n}\in S^{n}A^{n}$ is transmitted over the channel. The transmission rate of this scheme is R=k/n bit/1-D.

Since symbol-level shaping strategies determine m−1 amplitude bits prior to FEC encoding, they can only be combined with FEC code rates $R_{c}\geq \left(m-1\right)/m$. To employ lower FEC code rates $R_{c}<\left(m-1\right)/m$, bit-level shaping strategies which only determine a subset of m−1 amplitude bits should be employed as in [51,57,73]. To use a higher FEC code rate $R_{c}>\left(m-1\right)/m$, a modified PAS architecture is proposed in [28] as shown in Figure 3 (bottom). The code rate in this scheme is $R_{c}=\left(m-1+\gamma \right)/m$ where $\gamma =R_{c}m-\left(m-1\right)$ specifies the number of extra data bits that will be transmitted per symbol. In this modified structure, in addition to the n(m−1) bit output of the shaper, extra γn information bits ${\tilde{u}}^{\gamma n}$ are fed to the FEC code which is now specified by an n(1−γ)-by-nm parity-check matrix P. The (1−γ)n bit parity output of the FEC code is then multiplexed with the uniform bits ${\tilde{u}}^{\gamma n}$ to form an *n*-bit sequence that will select the signs $s^{n}$. The transmission rate of this scheme is R=k/n+γ bit/1-D.

**Example** **2** (Shaping, FEC and transmission rates in PAS)**.** *Consider the PAS architecture with 8-ASK, a rate $R_{c}=5/6$ FEC code, and a target rate R=2.25 bit/1-D. The rate of the extra data that will be carried in the signs of the channel inputs is $\gamma =R_{c}m-\left(m-1\right)=0.5$ bit/1-D. Therefore, the rate of the amplitude shaper should be k/n=R−γ=1.75 bit/1-D. If the length of the FEC code is $n_{c}=648$ bits, the blocklength is $n=n_{c}/m=216$ real symbols. Then the output set of the amplitude shaper must consist of at least $2^{k}=2^{216\cdot 1.75}=2^{378}$ sequences.*

### 3.3. PAS Receiver

At the receiver, the log-likelihood ratio (LLR) $L_{j}\left(i\right)$ of the *j*th bit in the *i*th transmitted symbol is computed by a soft demapper as

(11) $$\begin{array}{c} L_{j}\left(i\right)=\log\left(\frac{\sum_{x\in X_{j,0}}P_{X}\left(x\right)P_{Y|X}\left(y_{i}|x\right)}{\sum_{x\in X_{j,1}}P_{X}\left(x\right)P_{Y|X}\left(y_{i}|x\right)}\right) \end{array}$$

for j=1,2,⋯,m and i=1,2⋯,n, where $X_{j,u}$ denotes the set of x∈X which have $b_{j}=u$ in their binary labels for u∈{0,1}. We emphasize that the nonuniform a-priori information on the symbols is used in (11). Instead of symbol-wise probabilities $P_{X}\left(x\right)$, bit-wise probabilities $P_{B_{j}}\left(b_{j}\right)$ for j=1,2,⋯,m can be used to compute (11) as in (Equation (60) in [28]) or (Equation (3.29-32) in [8]). Then a bit-metric decoder uses the LLRs in (11) as the decoding metrics, i.e., treats different coded bits in a given symbol as independent [7], and estimates the bits that were encoded by the FEC code. In the case of uniform signaling, these bits are the estimates of the information bits. For the PAS architecture shown in Figure 3 (middle), the output of the decoder consists of the estimates of the amplitude bits. Then these are mapped back to the information bit estimates using the inverse functions of the blocks in the shaper (green box), i.e., the corresponding bit-to-amplitude mapper followed by the corresponding amplitude deshaper. In addition to this, for the PAS architecture shown in Figure 3 (bottom), the decoder also outputs the estimates of the γn extra data bits which were used as some of the signs. A bit-metric decoder achieves the rate $R_{\mathrm{BMD}}$ for any input distribution $P_{X}\left(x\right)$,

(12) $$\begin{array}{ccc} R_{\mathrm{BMD}} & = & {\left[H\left(X\right)-\sum_{j=1}^{m}H\left(B_{j}|Y\right)\right]}^{+} \end{array}$$

where ${\left[\cdot \right]}^{+}=\max\left\{0,\cdot \right\}$. In [84,85], the achievability of (12) is derived using random coding arguments based on strong typicality (Chapter 1 in [86]). Later in (Lemma 1 in [87]), it was shown that (12) is an instance of the LM rate [41,88]. AIRs of PAS have been studied in [80,89,90,91]. It is demonstrated that the mutual information I(X;Y) and the rate $R_{\mathrm{BMD}}$ in (12) are achievable with PAS using symbol-metric and bit-metric decoding, respectively.

### 3.4. Selection of Parameters for PAS

In this section, we study the optimum shaping and FEC coding rates for PAS using AIRs. Therefore, we consider the case where n→∞ which implies that k/n=H(A) for asymptotically optimum shaping architectures from (9), and consequently, R=H(A)+γ.

**Figure 4:**
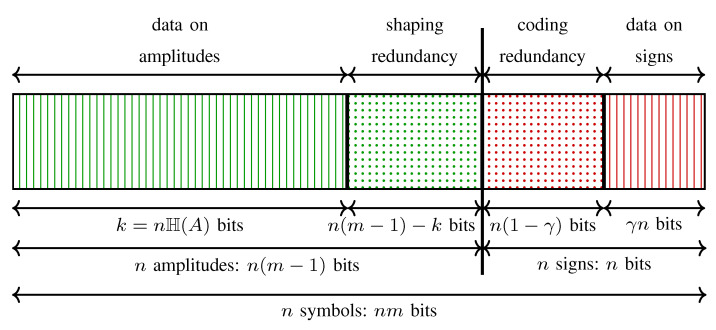
Content of a channel input sequence produced by PAS.

In the PAS architecture, to obtain a target rate R=H(A)+γ using the $2^{m}$-ASK constellation, a total of n(m−R) redundancy bits are added to a channel input sequence by shaping and coding operations combined. Shaping is responsible for n(m−1−H(A)) redundant bits whereas coding adds n(H(A)+1−R) bits. This is illustrated in Figure 4 where the content of a channel input sequence produced by the generalized PAS architecture of Figure 3 (bottom) is shown. The striped areas represent the information carried in signs (red) which is γn bits, and in amplitudes (green) which is k=nH(A) bits. Dotted areas show the redundant bits in a sequence. When γ=0, i.e., $R_{c}=\left(m-1\right)/m$, all signs are selected by redundancy bits and thus, the striped red area in Figure 4 vanishes. When H(A)=m−1, the amplitudes are uniformly distributed, i.e., there is no shaping, and thus, the dotted green area in Figure 4 disappears. We note that a similar illustration was provided for a single ASK symbol in (Figure 9 in [55]). In Table 1, the content of a sequence at the output of a PAS transmitter (in accordance with Figure 4) is tabulated for Example 2 where n=216.

When the input is constrained to be MB-distributed, H(X)=H(A)+1 can be used as a design parameter which tunes the balance between shaping and coding redundancies at a fixed rate *R*. More specifically, the entropy H(A) of the MB distribution (2) is controlled by λ. Thus by changing λ, the amount of shaping redundancy in an amplitude can be adjusted. The question is then how to choose the optimum λ. Following Wachsmann, Fischer and Huber [2,92], we use the gap to capacity (normalized SNR), which is defined as

(13) $$\begin{array}{c} \Delta \mathrm{SNR}=\frac{\mathrm{required}\;\mathrm{SNR}\;\mathrm{such}\;\mathrm{that}\;R_{\mathrm{BMD}}=R}{2^{2R}-1} \end{array}$$

as the metric to be minimized when searching for the optimum MB distribution for a fixed rate *R* and constellation size $2^{m}$. In general, the gap-to-capacity can be computed for any parametric family of distributions. Here we only consider the MB distributions since they have been shown to perform very close to the capacity of ASK constellations over the AWGN channel and maximize the energy efficiency [21]. The numerator in (13) is the SNR value at which $R_{\mathrm{BMD}}=R$ for a given $P_{X}$, and the denominator is the SNR value at which the capacity C=R. We note that instead of the MI in (Equation (55) in [2]), we now use the BMD rate of (12). Observing from Figure 4 that 1−γ=1−(R−H(A)), the rate of the FEC code that should be employed in PAS to obtain a transmission rate *R* for a given constellation entropy H(X) is given by

(14) $$\begin{array}{c} R_{c}=\frac{m-1+\gamma }{m}=\frac{m+R-H(A)-1}{m}=\frac{m+R-H(X)}{m}. \end{array}$$

**Figure 5:**
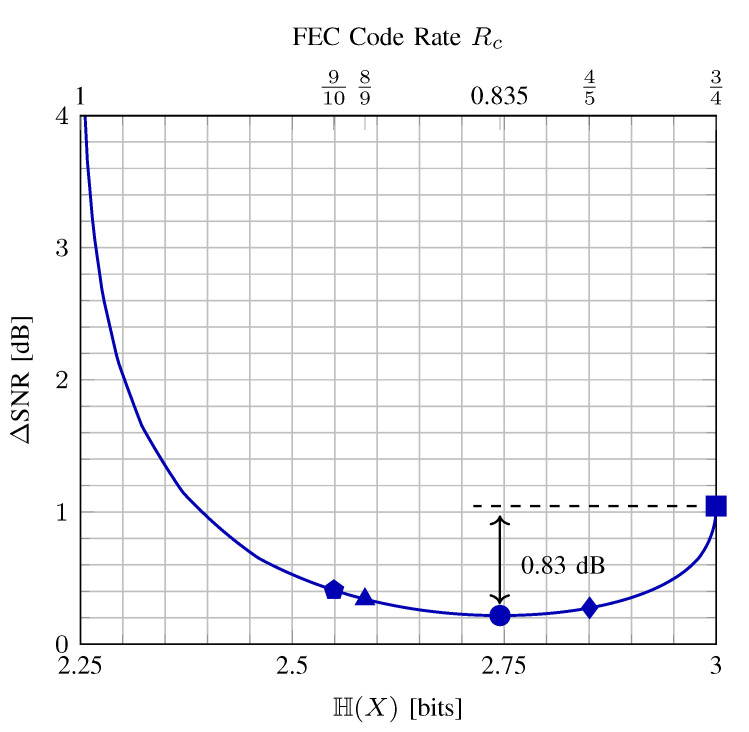
Channel input entropy vs. gap-to-capacity for 8-ASK at the target rate of R=2.25 bit/1-D. The x-axis above shows the corresponding FEC code rates.

**Example** **3** (Optimal PAS parameters)**.** *In Figure 5, the entropy H(X) of an input X with |X|/2=4 MB-distributed amplitude levels (i.e., 8-ASK) vs. ΔSNR is plotted for R=2.25 bit/1-D. On the top horizontal axis, the corresponding FEC code rates (14) are also shown. The rightmost point (indicated by a square) corresponds to uniform signaling where the target rate of 2.25 bit/1-D is obtained by using a FEC code of rate $R_{c}=R/m=3/4$. In this trivial case, all 0.75 bits of redundancy are added by the coding operation, and the gap to capacity ΔSNR is 1.04 dB. The leftmost part of the curve where H(X) goes to R belongs to the uncoded signaling case, i.e., $R_{c}=1$, where R is attained by shaping the constellation such that H(X)=R. Here ΔSNR is infinite since without coding, reliable communication is only possible over a noiseless channel. The minimum ΔSNR in Figure 5 is obtained with H(X)=2.745, which corresponds to $R_{c}=0.835$ from (14). In IEEE DVB-S2 [77] and 802.11 [78], the code rate that is closest to 0.835 is 5/6≈0.833. Accordingly, the best performance is expected to be provided with rate-5/6 FEC code, with an SNR gain over uniform that amounts according to this analysis to 0.83 dB. This will be confirmed by the numerical simulations presented in Section 5.3.*

## 4. Distribution Matching and Sphere Shaping Architectures

This section gives an overview of various shaping architectures that are compatible with the PAS framework. We focus on constructive methods, i.e., the direct use of a LUT for shaping or deshaping is not considered herein. Also, only fixed-length schemes are considered.

### 4.1. Distribution Matching Architectures (Direct Method)

In the following, an overview of distribution matching architectures and algorithms is given. The difference between these two aspects was discussed in Section 2.4. All of the following schemes have in common that a certain probability mass function (PMF) is targeted explicitly. For finite-length DM, this means that some quantization might be required as to achieve an integer-valued composition. Possible quantization rules include a simple rounding operation (Section V-A2 in [28]), or minimizing the Kullback-Leibler divergence [93]. We note that neither of these approaches is necessarily optimal in achieving the maximum information rate for a given *n* and channel law.

**Remark** **1** (On the validity of targeting the capacity-achieving distribution at finite blocklengths). *As discussed earlier in Section 2.2, MB-distributed ASK channel inputs do not maximize the AIR for the AWGN channel. Furthermore, the observation that the loss in AIR resulting from using a MB distribution instead of the AIR-maximizing distribution is negligible is only valid for asymptotically large signaling blocklengths. Accordingly, one can choose a target distribution that depends on the blocklength which may result in improved performance. Nevertheless, MB distributions have frequently been considered for shaping ASK constellations for finite (and short) blocklengths in the literature, and we take the same approach in this study.*

**Figure 6:**
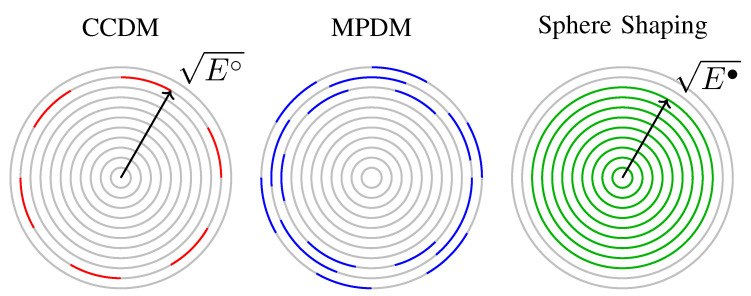
The illustration of the employed *n*-dimensional signal points by CCDM (**left**), MPDM (**middle**) and SpSh (**right**). Each circle represents an *n*-dimensional shell. Darker portions of the shells indicate the signal points on them which are utilized by the corresponding shaping approach.

CCDM has been proposed in [52] and it was used as the amplitude shaping architecture for PAS in [28]. We speak of constant composition if all matcher output sequences are permutations of a particular base sequence, which is typically described by the composition *C* stating the number of occurrences of each amplitude. The number of output sequences of the corresponding matcher, i.e., the cardinality of the shaping set $A^{\circ }\subseteq A^{n}$, is given by the multinomial coefficient $\mathrm{MC}\;\left(C\right)$, as defined in (5). Each amplitude sequence in $A^{\circ }$ has the same energy $E^{\circ }$, and consequently, they all are located on the *n*-shell of squared radius $E^{\circ }$ as shown in Figure 6.

**Example** **4** (CCDM)**.** *We consider the target PMF $P_{A}=\left[0.4378,0.3212,0.1728,0.0682\right]$ over A={1,3,5,7} with H(A)=1.75 bit. Combined with rate-5/6 FEC coding in PAS framework, an amplitude shaper with this entropy corresponds to a transmission rate of R=2.25 bit/1-D from (14), which is a typical target rate with 8-ASK. The composition that is obtained for n=216 with the quantization rule proposed in (Algorithm 2 in [93]) is C=[95,69,37,15]. The shaping rate (7) of the matcher that produces sequences with composition C is $R_{s}=1.6991$ bit/1-D. The input length (8) of this matcher is k=367 bits.*

MPDM has been proposed in [55] as an extension to CCDM that lifts the constant-composition principle. MPDM is based on the idea that the target composition *C* need not be achieved in each output sequence; rather, it is sufficient if the ensemble average over all sequences gives the target composition. Considering the example of pairwise partitioning in [55], this means that each composition has a complement, both with the same number of occurrences, such that their average is the target composition. There are, however, no known constructive algorithm for this variable-composition mapping problem. This is circumvented by reducing the number of unique sequences of each composition to be a power of two, which can come at the expense of some small rate loss. This additional constraint enables Huffman coding on the compositions, i.e., we can build a tree where a variable-length prefix determines the node and thus, the composition to be used. The remaining binary payload is mapped with conventional CCDM techniques. Note that the prefix and payload length are balanced such that the overall shaping operation is fixed-length. It has been shown in [55] that compared to CCDM, pairwise MPDM with such a tree structure gives an approximately fourfold reduction in required blocklength to obtain the same information rate. In addition, pairwise MPDM has also been demonstrated to give significant achievable rate and SNR improvements for a fixed blocklength over CCDM for the AWGN channel [94] and the optical fiber channel [95].

**Example** **5** (MPDM)**.** *We consider the same target PMF and n=216 as in Example 4. Pairwise MPDM with tree structure utilizes 945 compositions whose average is again [95,69,37,15]. The shaping rate (7) of the matcher that produces sequences with these compositions is $R_{s}=1.7315$ bit/1-D. The corresponding input length (8) is k=374 which is 7 bits more than that of CCDM which is a 1.9% rate increase.*

CCDM has initially been realized with AC, which is sequential in the input length, i.e., at most *k* serial operations must be carried out for shaping, and *n* for deshaping (Note that this describes the worst-case serialism if the DM operation cannot be terminated early, which could be the case when the remainder of the output sequence follows at some point with certainty. Also, this metric does not incorporate the complexity of the computations inside each step as discussed in Section 6). Since the serialism of the AC method can be challenging to achieve for high-throughput CCDM operation, means to run several DMs in parallel have been proposed. For BL-DM [57] or PDM [51] where the target distribution is a product distribution, the parallelization factor is $\log_{2}n_{a}=m-1$ since one binary-alphabet DM is used for each bit level. This approach has been numerically shown to have reduced rate loss compared to a single nonbinary DM, yet comes at the expense of having the DM output limited to compositions that are generated from a product distribution. In [58], a different parallelization technique has been proposed, which operates on amplitudes rather than on bit levels. For each of the $n_{a}-1$ out of $n_{a}$ amplitudes, a binary-alphabet DM is operated in parallel, with the first DM determining the position of the first amplitude, the second DM where to position the second amplitude within those positions that have not been occupied by the preceding, i.e., the first, amplitude. These DM operations can be run in parallel and only the final step of combining the subsequences into the nonbinary output sequence is sequential. We note that both bit-level DM and parallel-amplitude DM are compatible with MPDM.

The schemes discussed in the preceding paragraphs can be considered as extensions to the CCDM architecture that either nest various CCDMs for improved performance (MPDM) or transform a nonbinary CCDM into several binary CCDMs to achieve a larger parallelization (bit-level and parallel-amplitude DM). In [58], subset ranking (SR) has been proposed as an alternative to the conventional AC algorithm for CCDM as shown in the bottom layer of Figure 1. SR solves the CCDM indexing problem by representing a binary-alphabet sequence as a constant-order subset that determines the position of either binary symbol. For a given sorting, such as lexicographical, the rank of such a subset is found by “enumerating” all preceding sequences which is used for source coding in [96,97], and for shaping in [22,65]. This mapping from sequence to (binary) rank is called unranking in the combinatorics literature and acts as deshaping. The ranking operation from bits to sequence is shaping. The advantage of SR over AC is that the number of serial operations is significantly reduced (Section V in [58]).

### 4.2. Sphere Shaping Architecture (Indirect Method)

In this section, a review of SpSh algorithms is provided. All ensuing algorithms target a certain rate, i.e., the number of unique output sequences, rather than a PMF. To this end, for a given A, *n* and target *k*, the maximum-energy constraint $E^{•}$ is selected as the minimum value such that the corresponding set $A^{•}$, as defined in (4), satisfies $|A^{•}|\geq 2^{k}$. This set consists of all $2^{m}$-ASK amplitude lattice points on the surface of or inside the *n*-sphere of square radius $E^{•}$ as shown in Figure 6. (We use “$2^{m}$-ASK amplitude lattice” for the *n*-fold Cartesian product of $\left\{1,3,\cdots ,2^{m}-1\right\}$ with itself, i.e., $A^{n}$.) We note that possible sequence energy values for these points, i.e., squared radii of the *n*-dimensional shells that the sequences are located on, are $\left\{n,n+8,\cdots ,E^{•}\right\}$, and the maximum-energy constraint can be written as

(15) $$\begin{array}{c} E^{•}=n+8\left(L-1\right) \end{array}$$

where *L* denotes the number of these *n*-dimensional shells (Since the AWGN channel capacity in (1) is computed starting with the assumption that the signal points outside the *n*-sphere of squared radius nP cannot be transmitted, i.e., the average power constraint, one could argue that referring to “sphere shaping” as the “indirect” method is inaccurate. However, this constraint only prevents the use of the signal points outside the sphere, and does not prescribe the use of all the ones inside. Therefore, we choose to follow the nomenclature introduced by Calderbank and Ozarow in [19], which formulates the problem as “achieving the capacity of a channel of which the capacity-achieving input distribution is Gaussian”).

**Remark** **2.** We see from the sphere-hardening result discussed, e.g., by Wozencraft and Jacobs in (Section 5.5 in [98]), that $E^{•}\approx nE$ for large n. Following Laroia et al. (Section III-A [23]) and approximating the required average energy to transmit R bit/1-D by $c2^{2R}$, we can write $E^{•}\approx nc2^{2R}$ where c is some constant. Therefore, for a fixed rate R and for large n, we assume that L in (15) depends linearly on n.

**Example** **6** (Sphere shaping)**.** *The sphere shaping set $A^{•}\subset A^{n}$ for the parameters n=64, A={1,3,5,7} and $E^{•}=768$, i.e., L=89, has the shaping rate $R_{s}=1.7538$ bit/1-D. The input length of the corresponding amplitude shaper is k=112 bits. The average PMF is $P_{A}\left(a\right)=\left[0.42,0.32,0.18,0.08\right]$ over A, where the average energy per dimension is E=11.6316.*

In the following, we explain two different algorithms to realize SpSh: Enumerative sphere shaping (ESS) and shell mapping (SM). Provided with identical parameters, these two address the same set $A^{•}$ of sequences where the difference is in the bits-to-amplitudes mapping.

ESS starts from the assumption that the energy-bounded amplitude sequences, i.e., $a^{n}\in A^{•}$, can be ordered lexicographically. Therefore, the index of an amplitude sequence is defined to be the number of sequences which are lexicographically smaller. To represent *n*-amplitude sequences inside a sphere, an energy-bounded enumerative amplitude trellis is constructed (Section III-B in [48]). Operating on this enumerative trellis, *n*-step recursive algorithms are devised to realize the lexicographical index-sequence mapping in an efficient manner [22,65]. These algorithms demand the storage of an (n+1)-by-*L* matrix, i.e., the trellis, where each element can be up to (k+1)-bit long. The required storage and computational complexity of ESS is discussed in Section 6.

Another way of ordering *n*-amplitude sequences inside a sphere is to sort them based on their energy, i.e., based on the index of the *n*-dimensional shell that they are located on. Sequences on the same shell can be sorted, e.g., lexicographically. To this end, a trellis which is different from that of ESS is constructed [23,65]. Based on this trellis, two different indexing algorithms are proposed in [23]. The first one, (Algorithm 1 in [23]), which was proposed around the same time as ESS [22], has performance and complexity similar to ESS. The second one, (Algorithm 2 in [23]), which is the well-known shell mapping (SM), is based on the divide-and-conquer (D&C) principle, and enables a tradeoff between the computational and storage complexities (Section 4.3 in [10]). The D&C principle was used to enumerate sequences from the Leech lattice earlier in [59]. The basic principle is to successively divide an *n*-dimensional indexing problem into two n/2-dimensional problems, creating a $\log_{2}n$-step operation. Consequently, SM demands the storage of a $\left(\log_{2}n+1\right)$-by-*L* matrix, where each element is again at most (k+1)-bit long. The required storage and computational complexity of SM is discussed in Section 6.

In their initial proposals, shaping matrices of ESS [22], SM [23] and (Algorithm 1 in [23]) are computed with full-precision (FP). To decrease the required storage for these algorithms, a bounded-precision (BP) implementation method is proposed in [65]. The idea is that any number can be expressed in base-2 as $m\cdot 2^{p}$. Here *m* and *p* are called the mantissa and the exponent, stored using $n_{m}$ and $n_{p}$ bits, respectively. Then each number in a shaping matrix, i.e., in the trellis, is rounded down to $n_{m}$ bits after its computation, and stored in the form (m,p). The invertibility of ESS and SM functions is preserved with this approach [65]. We note that the BP implementation can also be used to realize (Algorithm 1 in [23]). The BP implementation decreases the memory required to store an element of the shaping trellis from (k+1) bits to $n_{m}+n_{p}$ bits. Typical values of $n_{m}$ and $n_{p}$ are a few bytes. The required storage and the computational complexity of BP implementation is discussed in Section 6. The disadvantage of this approximation is that the numbers in the trellis, and thus, the number of represented sequences decreases, causing a rate loss. However, this rate loss is shown to be upper-bounded by $-\log_{2}\left(1-2^{1-n_{m}}\right)$ bit/1-D [65].

**Example** **7** (Bounded-precision rate loss)**.** *If the shaping set $A^{•}$ in Example 6 is constructed with BP using $n_{m}=9$ bit mantissas and $n_{p}=7$ bit exponents, the resulting rate loss is upper-bounded by 0.0056 bit/1-D. For ESS and SM, the actual rate losses are 0.0021 and 0.0003 bit/1-D, respectively. Since the shaping rate with FP was $R_{s}=1.7538$, these rate losses keep $R_{s}>1.75$, and consequently, keep k=112. Therefore, we claim that when more than a few bytes are used to store mantissas, BP rate loss is smaller than the loss due to the rounding operation in (8). Consequently, the operational rate k/n is not affected. However, the required memory to store an element of the shaping matrix drops from (k+1)=113 bits to $n_{m}+n_{p}=16$ bits.*

Both ESS and SM index the same set of sequences for fixed *n*, A and $E^{•}$. However, the way they order sequences is different. Due to the round-down operation in (8), only the sequences with indices smaller than $2^{k}$ are actually utilized. The remaining ones, i.e., the ones at the end of the ordered list, are unused. For SM, all these sequences have the highest possible energy $E^{•}$. On the other hand for ESS, these sequences are at the end of the lexicographical list and are not necessarily from the outermost shell. Thus operationally, average symbol energy of SM is no greater than that of ESS, for a fixed set of parameters. This difference could be important for ultra short blocklengths, however, for blocklengths larger than a few dozens, it becomes insignificant. Furthermore, as discussed in [99], by simply removing some connections from the shaping trellis, it is possible to force the discarded sequences to be from the outermost shell for ESS as well.

### 4.3. Geometric Interpretation of the Shaping Architectures

Output sequences of CCDM have a fixed composition and thus, all have the same sequence energy nE, i.e., they are located on the *n*-dimensional shell of squared radius $E^{\circ }=nE$. We note that there are multiple compositions that lead to the same sequence energy and thus, the corresponding shell is only partially utilized by CCDM, as shown in Figure 6 (left). With multiple compositions at its output, MPDM makes use of multiple partly filled *n*-shells, as in Figure 6 (middle). The average symbol energy as well as the squared radius of the outermost shell that is utilized by MPDM depend on the actual set of considered compositions. Finally, *n*-sphere shaping employs all sequences inside the *n*-dimensional sphere of squared radius $E^{•}$, as shown in Figure 6 (right). Note that for simplicity, we have in this explanation neglected the constraint that any practical binary scheme can only address a power-of-two number of shaped sequences. When all three approaches enclose the same number of sequences at a fixed *n*, their average energy as in (6) satisfy $E_{\mathrm{ccdm}}\geq E_{\mathrm{mpdm}}\geq E_{\mathrm{spsh}}$. Therefore, at any blocklength, SpSh makes use of the set of sequences having the least average energy and thus, it is the most energy-efficient scheme. This observation will later be confirmed by the rate loss analysis in Section 5.1.

## 5. Performance Comparison

This section studies the performance of the shaping architectures explained in Section 4. The used metrics are (i) finite-length rate loss at a fixed blocklength *n*, (ii) finite-length information rates for BMD, and (iii) frame error rate (FER). Communication systems with limitations on peak power or peak-to-average power ratio are not considered here.

### 5.1. Rate Loss Analysis

**Figure 7:**
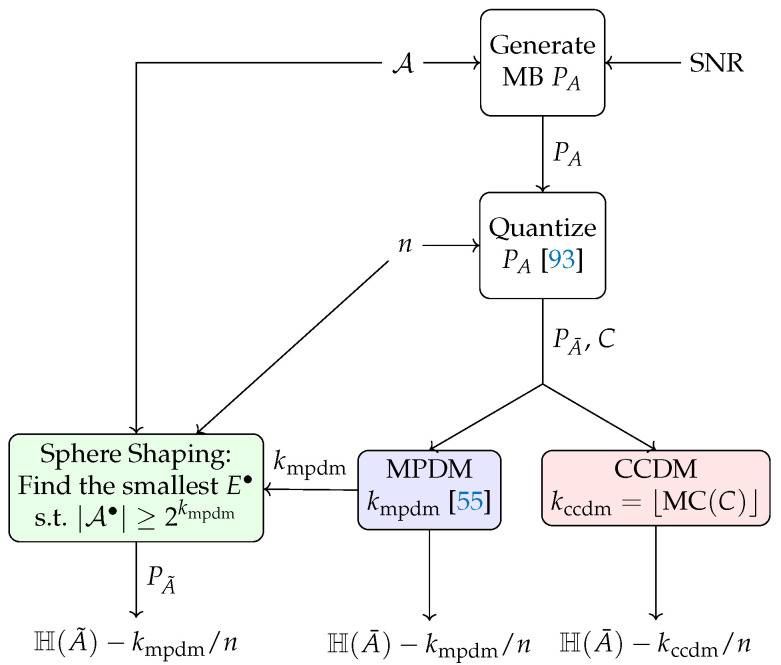
Flowchart for the computation of rate loss for CCDM, MPDM and SpSh.

The methodology of computing the rate loss for DM and SpSh schemes in a fair manner is illustrated in Figure 7. For the DM schemes of Section 4.1, the following steps are carried out in order to obtain the rate loss for a particular *n*. First, the target distribution $P_{A}$ (and thus the modulation order $2^{m}$) is fixed. The target distribution is MB, optimized for a particular SNR. We then quantize $P_{A}$ to $P_{\bar{A}}$ to get the integer-valued target composition $C=nP_{\bar{A}}$, where the quantization criterion is to minimize the Kullback-Leibler divergence between $P_{A}$ and $P_{\bar{A}}$ [93]. For CCDM, $k=\left\lfloor \log_{2}\mathrm{MC}\;\left(C\right)\right\rfloor$ bits can be addressed where the multinomial coefficient $\mathrm{MC}\;\left(\cdot \right)$ is as defined in (5). For nonconstant composition DMs such as MPDM [55], the number of addressable bits *k* depends on the addressable bits of all constituent compositions, considering the specific constraints of the DM construction such as pairwise partitioning (Section III-A in [55]). The rate loss is finally computed as $R_{\mathrm{loss}}=H\left(\bar{A}\right)-k/n$, as defined in (10).

For the SpSh schemes of Section 4.2, the approach must be different since it is not possible to explicitly target a certain distribution or composition. From the above methodology, we obtain the number of input bits $k_{\mathrm{mpdm}}$ for MPDM at a given *n*. For each *n*, we find the smallest $E^{•}$, i.e., the squared radius of the sphere, such that the number of sequences inside the *n*-sphere $A^{•}$ satisfies $\log_{2}\left(\right|A^{•}\left|\right)\geq k_{\mathrm{mpdm}}$. We compute the average distribution $P_{\tilde{A}}$ (Equation (17) in [48]). The rate loss is again obtained as $R_{\mathrm{loss}}=H\left(\tilde{A}\right)-k/n$. For SpSh schemes, the input length $k_{\mathrm{ccdm}}$ of CCDM can also be targeted during the rate loss computation. However, we prefer to use input length of MPDM since in general, $k_{\mathrm{mpdm}}\geq k_{\mathrm{ccdm}}$.

**Example** **8** (Rate loss comparison)**.** *We consider the target distribution $P_{A}=\left[0.438,0.321,0.173,0.068\right]$ with entropy H(A)=1.75. The n-type distribution that has the minimum informational divergence from $P_{A}$ for n=216 is $P_{\bar{A}}=\left[0.440,0.320,0.171,0.069\right]$. The corresponding composition is C=[95,69,37,15]. Starting with the same target distribution, i.e., with the same composition, the number of compositions that are employed by MPDM is 945 (Section III in [55]). Since MPDM’s set of compositions consists of pairs whose average is C, the average distribution $P_{\bar{A}}$, its entropy $H(\bar{A})$ and the average symbol energy E are the same as CCDM’s. The smallest $E^{•}$ that gives $|A^{•}|\geq 2^{k}$ is $E^{•}=2376$ where k is the input length of MPDM. The corresponding average distribution is $P_{\tilde{A}}=\left[0.439,0.322,0.172,0.067\right]$. Table 2 shows the input length k, average symbol energy E and rate loss $R_{\mathrm{loss}}$ of CCDM, MPDM and SpSh for these parameters. We see that MPDM is able to address a larger set of sequences than CCDM, leading to a 7 bit increase in the input length. Since the corresponding average distributions are the same, this is reflected as a decrease in rate loss. Then starting with the same target k, SpSh employs a set of sequences with smaller average energy. This is also translated to a decrease in rate loss as shown in Table 2.*

**Remark** **3** (Targeting a rate with DM)**.** *Example 8 shows that when the entropy of the target distribution is taken to be the target rate k/n (1.75 bit/1-D in Example 8), CCDM and MPDM are not able to obtain $2^{k}$ sequences. This is due to the inevitable nonzero rate loss of the DM schemes for finite blocklengths. For such cases, we increase the SNR that the target distribution is optimized for, until we obtain $2^{k}$ output sequences for the DM schemes.*

**Figure 8:**
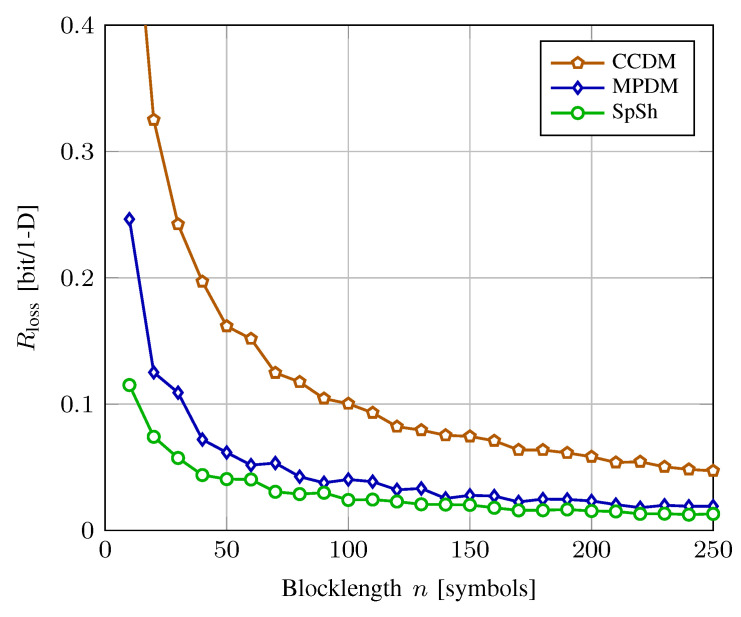
Rate loss vs. blocklength for various shaping architectures.

Figure 8 shows rate loss vs. blocklength for CCDM, MPDM, and SpSh. The target distribution is the same as Example 8. The target *k* for ESS is the number of bits achieved by MPDM at each *n*. We observe that MPDM and SpSh clearly outperform CCDM. Furthermore, the more efficient signal space usage of SpSh becomes particularly apparent at very short blocklengths. Here we note that in Figure 8, we used the distribution averaged over all sequences inside an *n*-sphere, i.e., sphere distribution, to compute the rate loss for SpSh. This relies on the fact that the sphere distribution is an accurate approximation for the distribution averaged over the $2^{k}$ sequences that are actually transmitted, i.e., operational distribution. The operational distribution and the actual rate loss depend on the SpSh algorithm that is employed. However, the difference in rate loss for different SpSh algorithms is only significant for ultra short blocklengths. As an example, at n=8 and k/n=1.75 bit/1-D with 8-ASK, the rate loss computed using the sphere distribution is 0.0924 bit/1-D, while the actual rate losses are 0.0912, 0.0908 and 0.0907 for ESS, (Algorithm 1 in [23]) and SM, respectively. The operational distribution of SM can be computed using the ideas presented in [100].

### 5.2. Achievable Information Rates

Here, we numerically study the information rates of CCDM, MPDM, and SpSh in the finite blocklength regime. As the figure of merit, the finite-length information rate for BMD is used as defined in (Equation (15) in [55]):

(16) $$\begin{array}{c} {\mathrm{AIR}}_{n}=R_{\mathrm{BMD}}-R_{\mathrm{loss}}. \end{array}$$

Here $R_{\mathrm{BMD}}$ and $R_{\mathrm{loss}}$ are as defined in (12) and (10). We note that (16) converges to (12) for asymptotically optimum shaping architectures when n→∞. Although the finite length information rate ${\mathrm{AIR}}_{n}$ in (16) is not an “achievable” rate in the strict sense, it has been employed to compare CCDM and ESS for the optical fibre channel in [61,62,63]. We note here that (16) is an instance of the rate expression (Equation (1) in [101]) provided for the *layered PS* architecture. We refer the reader to (footnote 3 in [61]) for a discussion on the derivation of (16) from (Equation (1) in [101]).

**Figure 9:**
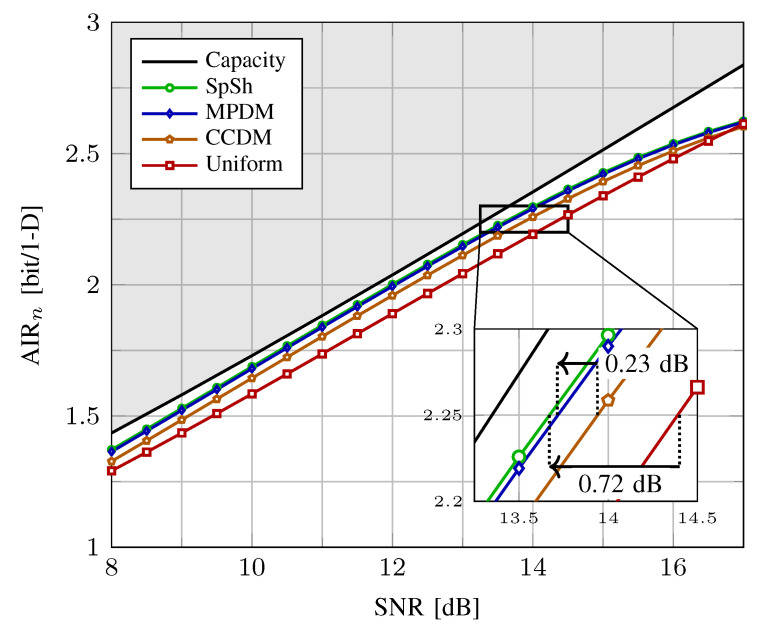
Finite-length information rate vs. SNR for various shaping architectures at n=216.

In Figure 9, ${\mathrm{AIR}}_{n}$ (in bit/1-D) is shown versus SNR (in dB) for CCDM, MPDM and SpSh with 8-ASK. We use shaping blocks of length n=216, which is compatible to the $n_{c}=648$-bit LDPC codes of IEEE 802.11 [78] that will be employed in PAS in subsequent sections. Shaping algorithms operate at a rate of k/n=1.75 bit/1-D, i.e., *k* is set to 378 bits. We note that this means we plotted the curves for fixed distributions and did not optimize them at each SNR, unlike (Figure 4 in [28]) or (Figure 5 in [55]). For comparison, the Shannnon capacity $\frac{1}{2}\log\left(1+\mathrm{SNR}\right)$ and the GMI for uniform 8-ASK are also plotted. We observe that SpSh and MPDM close most of the shaping gap. From the inset figure, we see that SpSh and MPDM are roughly 0.72 dB more SNR-efficient than uniform signaling at rate R=2.25 bit/1-D. We note that this rate corresponds to γ=R−k/n=0.5, and thus, $R_{c}=5/6$ in the PAS context. As a reference, the maximum possible capacity gain for 8-ASK at this rate that can be obtained by using the optimum MB distribution was shown to be 0.83 dB in Figure 5. The remaining gap of 0.11 dB is due to the finite blocklength nature of the shaping architectures. Finally, from the inset of Figure 9, we see that SpSh and MPDM are approximately 0.23 dB more SNR-efficient than CCDM due to their energy-efficient use of the signal space.

We conclude from Figure 8 and Figure 9 that from a practical point of view, SpSh and MPDM perform almost the same at blocklengths larger than a few hundreds. Therefore, to make a choice among these at such values of *n*, required storage, computational complexity and latency of the algorithms that can be used to realize SpSh and MPDM should be considered. We will discuss these aspects of shaping algorithms in Section 6.

### 5.3. End-to-End Decoding Performance

In the following, the decoding performance of PAS is evaluated after transmission of 64-QAM over the AWGN channel. The BRGC in Figure 2 (bottom) is used for amplitude to bit mapping after shaping, and for symbol mapping after FEC encoding as shown in Figure 2 (top). Different transmission rates, and codeword length regimes of LDPC codes are considered. For each SNR, the simulations are run until at least 100 frame errors are observed. For the first case of long FEC codes, we use the LDPC codes from the DVB-S2 standard [77] with codeword length $n_{c}$ = 64,800 bits. In the case of short FEC codes, the LDPC codes from the IEEE 802.11 standard [78] with codeword length $n_{c}=648$ bits are used. Maximum 50 iterations are performed during the belief propagation decoding at the receiver.

For a fixed 1-D constellation size $M=2^{m}$, FEC code rate $R_{c}$ and target transmission rate *R*, we compute $\gamma =R_{c}m-\left(m-1\right)$ and accordingly, k/n=R−γ. Here, the total number of 1-D symbols in an $n_{c}$-bit FEC codeword is *n*. For DM algorithms working with A={1,3,5,7}, the AWGN-optimal MB PMFs at 10.7 and 14 dB SNR are quantized to obtain the integer composition based on [93] for the target rates 2 and 2.25 bit/1-D, respectively. For SpSh algorithms, $E^{•}$ is selected as the minimum value that satisfies $R_{s}\geq k/n$. Both ESS and SM are then implemented with full-precision.

**Figure 10:**
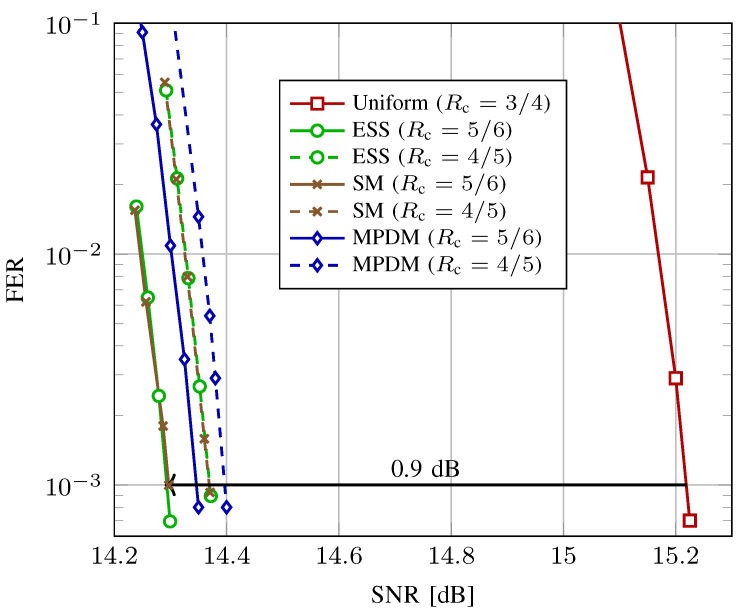
FER vs. SNR for 64-QAM at a transmission rate of 4.5 bit/2-D. Rate-$R_{c}$ DVB-S2 LDPC codes of length $n_{c}$ = 64,800 bits are used. All shaping schemes use a blocklength of n=180. At this shaping blocklength, each LDPC codeword consists of 120 shaped blocks.

Figure 10 shows the decoding performance with the LDPC codes from DVB-S2 for ESS, SM, MPDM, and uniform signaling at a transmission rate of 4.5 bits per complex channel use (bit/2-D). ESS, SM and MPDM, all of length n=180 amplitudes, use either the LDPC code of rate $R_{c}=5/6$ (solid curves) or rate $R_{c}=4/5$ (dashed lines). In order to achieve a transmission rate of 4.5 bit/2-D, the redundancy added by the shaping scheme is varied. For rate-5/6-coded signaling, γ=0.5 and thus, k/n=1.75 bit/1-D. For rate-4/5-coded signaling, γ=0.4 and thus, k/n=1.85 bit/1-D. For uniform 64-QAM, the FEC code rate is set to $R_{c}=3/4$. We observe for shaped schemes that the performance with FEC code of rate 5/6 is superior to rate 4/5 as predicted in Figure 5, for which the reasons are outlined in Section 3.4. Therefore, we will focus on $R_{c}=5/6$ in the following.

In Figure 10, at a FER of $10^{-3}$, SpSh and MPDM outperform uniform signaling by approximately 0.9 dB. We further note that ESS, SM and MPDM have very similar performance, with ESS and SM being approximately 0.05 dB more power-efficient than MPDM. This is in good agreement with the rate loss analysis of Figure 8 where also only a marginal improvement of the SpSh schemes over MPDM is found. Finally, as discussed in Section 5.1, there is no visible difference in performance for ESS and SM at n=180.

**Figure 11:**
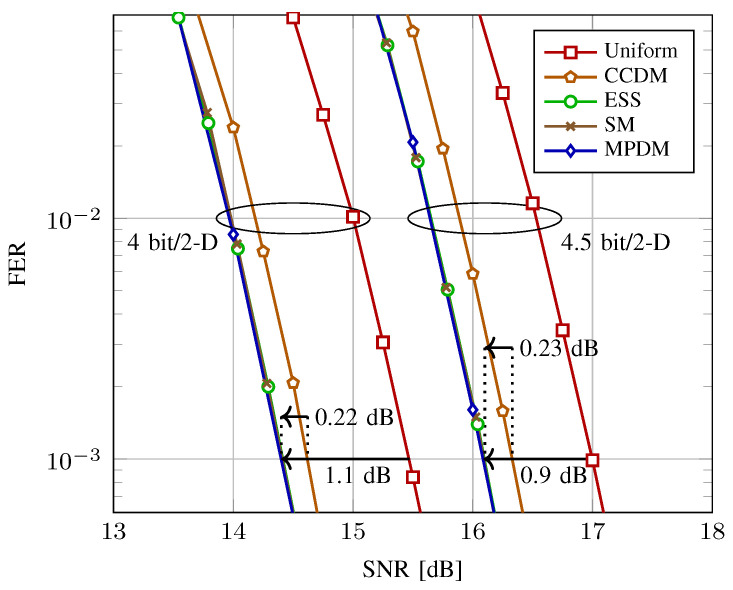
FER vs. SNR for 64-QAM at transmission rates of 4 and 4.5 bit/2-D. Rate-5/6 IEEE 802.11 LDPC codes of length $n_{c}=648$ bits are used for shaped signaling. All shaping schemes use a blocklength of n=216. At this shaping blocklength, each LDPC codeword consists of 1 shaped block.

For short LDPC codes, the shaping blocklength is set to n=216. In Figure 11, the decoding performance is shown at transmission rates of 4 and 4.5 bit/2-D. Uniform 64-QAM is encoded with LDPC codes of rate 2/3 and 3/4, respectively. For shaped signaling, the code rates that minimize ΔSNR for 64-QAM at rates 4 and 4.5 bit/2-D are computed to be $R_{c}\approx 0.79$ and 0.83 using (13), respectively. Therefore, being the closest available to these values, $R_{c}=5/6$ is used for shaped signaling.

As shown in Figure 11, at rates 4 and 4.5 bit/2-D, SpSh and MPDM perform similarly, and require 1.1 and 0.9 dB less SNR than uniform signaling to achieve a FER of $10^{-3}$, respectively. We further observe that they are 0.22 and 0.23 dB more power-efficient than CCDM at rates 4 and 4.5 bit/2-D, respectively. Since SpSh and MPDM perform almost identically for the considered shaping length, we believe the implementation aspects, which are discussed next, are of significant importance in the comparison between these architectures.

## 6. Approximate Complexity Discussion

In Section 5, we followed the conventional approach of comparing different shaping architectures by studying the blocklength that is required to obtain a certain shaping gain. While this is certainly a natural choice for analysing and comparing shaped systems, this approach inherently assumes that shorter blocks are always better, for instance because they have advantages regarding implementation. In the following, we comment on the implementation aspect by considering computational complexity, latency, and storage requirements.

An example where slightly longer blocklengths can be beneficial also from an implementation perspective is the parallel-amplitude (PA) architecture proposed in (Section III in [58]). By allowing a small additional rate loss, the throughput is increased significantly by using |A|−1 DMs in parallel. Furthermore, the serialism (and thus, the latency) of the subset ranking (SR) method of (Section IV in [58]) is smaller than AC-CCDM. It can thus be beneficial to make the blocks slightly larger than for conventional CCDM in order to facilitate implementation.

An interesting example where the selection of the shaping blocklength does not depend only on the complexity vs. shaping gain tradeoff is the nonlinear regime of the optical fibres. The authors of [61] recently found that shaping over shorter blocklengths increases the nonlinear tolerance, and thus, the effective SNR. Their claim is that when the complexity considerations are ignored, there is an optimum *n* that optimizes the balance between linear shaping gain and increased nonlinearity-tolerance.

Finally, we note that in this section, we restrict our attention to algorithmic implementation, while aspects related with hardware implementation such as throughput, clock frequency, and number of instances are outside the scope of this paper. For a detailed discussion on these concepts, shaping algorithms that are discussed in this work should actually be implemented as in [102,103,104,105].

### 6.1. Latency

In order to evaluate the latency of the discussed amplitude shaping algorithms, we use the concepts of “degree of serialism” and “parallelization factor” as defined in [58]. Degree of serialism is the number of loop iterations that are completed for shaping/deshaping operations. We stress that this quantity neglects the computational complexity of these iterations, and thus, the latency of the operations within each sequential processing step. Therefore, the degree of serialism can only serve as a rough indicator for latency. On the other hand, parallelization factor is the number of simultaneously possible executions of a process to complete shaping/deshaping operations.

AC, which can be employed to realize CCDM, is by nature a highly serial algorithm, and AC-CCDM has a serialism of *k* for matching and *n* for dematching [52]. SR-DM, which is an alternative to AC-CCDM in the binary-output case [58], has a serialism of $\min(n_{1},n-n_{1})$ and 1 for shaping and deshaping, respectively (In the SR-DM context, $\left[n_{1},n_{2}\right]$ is the composition of binary sequences at the output of the matcher).

In BL-DM [57] and PDM [51], a binary-output matcher is used for each of the $\log_{2}n_{a}=m-1$ amplitude bit levels to enable parallelization, and thus, the parallelization factor is $\log_{2}n_{a}$. As another attempt, PA-DM uses a binary-output matcher for $n_{a}-1$ of the $n_{a}$ amplitudes [58], and thus, the parallelization factor is $n_{a}-1$. A more detailed discussion on improving the parallelization of DM algorithms was provided in Section 4.1.

The shaping and deshaping algorithms of ESS [22] and (Algorithm 1 in [23]) have a serialism of *k* and *n*, respectively. On the other hand SM [23] operates based on the D&C principle as in [59], and therefore has a serialism of $\log_{2}n$ for deshaping. Table 3 summarizes the serialism of discussed shaping schemes.

### 6.2. Storage Requirements

AC-CCDM, which employs an extension of [53] to nonbinary-output, associates an interval in [0,1) to each binary input sequence and to each constant composition amplitude sequence (Section IV in [52]). In simplified terms, the final interval is computed by recursively splitting the initial interval into $n_{a}$ subintervals. The algorithm only requires the storage of the interval and the source statistics (i.e., the composition) which can be realized with logn bits (Here we assume that the memory required to store the interval is negligible, and roughly $\log_{2}n$ bits are enough to store the composition which consists of numbers that add up to *n*). Thus, we denote the storage complexity of AC-CCDM by O(logn). A similar reasoning can be used to determine the storage complexity of SR-DM (Section IV in [58]) which is also O(logn).

In MPDM, in addition to the requirements of the underlying CCDM algorithm, a composition is chosen based on a prefix of the binary input sequence. For this purpose, a prefix code and the corresponding Huffman tree is constructed (Section III-C in [55]). To store the binary-tree, a LUT can be used. The size of this table depends on the number of utilized compositions. For practical scenarios, the number of compositions is on the order of a few hundreds as shown in the following example.

**Example** **9** (MPDM, number of compositions)**.** *We consider A={1,3,5,7}, n=216 and target rates k/n=1.5 and 1.75 bit/1-D. To obtain these rates, MPDM uses 318 and 593 different compositions, respectively. Assuming that numbers in a composition can be stored using at most $\left\lceil \log_{2}n\right\rceil$ bits, at most 10,176 and 18,976 bits of memory are required to store the corresponding LUTs, respectively. Note that these are the parameters that are used for the simulations considered in Figure 11.*

FP implementations of ESS and (Algorithm 1 in [23]) require the storage of an *n*-by-*L* matrix where each element is at most $\left\lceil nR_{s}\right\rceil$-bits long. Therefore, following Remark 2, the storage complexity of these algorithms is $O(n^{3})$ for fixed $R_{s}$. FP SM can be realized by storing a $\log_{2}n$-by-*L* matrix [23], which has complexity $O(n^{2}\log n)$. We note here that these values are in alignment with (Table I in [23]).

**Example** **10** (FP SpSh, required storage)**.** *To realize ESS or (Algorithm 1 in [23]) for the setup in Example 6, at most $Ln\left\lceil nR_{s}\right\rceil =80.46$ kilobytes (kB) of memory is required. On the other hand for SM, at most $L\log_{2}n\left\lceil nR_{s}\right\rceil =7.54$ kB of memory should be allocated.*

**Remark** **4.** *To compute the required storage for SpSh in the BP case, we will assume that $n_{m}$ is independent of n. This assumption relies on the fact that the rate loss resulting from BP only depends on $n_{m}$ [65]. Thus, for a fixed rate loss, the required value of $n_{m}$ is independent of n. Expressing the number of bits to store the exponent as $n_{p}=\left\lceil \log_{2}\left(\left\lceil nR_{s}\right\rceil -n_{m}\right)\right\rceil$, we see that $n_{p}$ behaves as $\log_{2}n$ for a fixed $n_{m}$. We note here that for a fixed n, A and target k, the natural choice for $n_{m}$ is the smallest value that keeps the number of sequences at least $2^{k}$ [65].*

For the BP implementations of ESS, SM and (Algorithm 1 in [23]), each element of the stored shaping matrix is at most $\left(n_{m}+n_{p}\right)$-bit long [65]. Following Remark 4, the storage complexity of ESS and (Algorithm 1 in [23]) in the BP case is $O(n^{2}\log n)$. On the other hand the storage complexity of BP SM is $O(n\log^{2}n)$.

**Example** **11** (BP SpSh, required storage)**.** *To realize ESS or (Algorithm 1 in [23]) with $n_{m}=9$ and $n_{p}=7$ for the setup in Example 6, at most $Ln(n_{m}+n_{p})=11.39$ kB of memory is required. On the other hand, when implemented using $n_{m}=6$ and $n_{p}=7$, SM demands at most $L\log_{2}n\left(n_{m}+n_{p}\right)=0.87$ kB of memory. We note that the mantissa lengths $n_{m}$ are selected according to the discussion in Remark 4.*

In conclusion, we believe that storage requirements in the order of a few kBs are not critical for high-throughput operation, particularly in comparison to latency and complexity. Note that the required storage for BL-DM, PDM and PA-DM depends on the underlying algorithm.

### 6.3. Computational Complexity

To comment on the computational complexity of the amplitude shaping algorithms, we will mainly consider the number of required arithmetic operations or computations of binomial coefficients (BC). The caveat here is that this approach only gives a rough estimate since the complexity of an operation depends heavily on the specific case that it is executed in. As an example, the seemingly simple operation of comparing the sizes of two numbers can be computationally challenging for large numbers. On the other hand the notoriously expensive division operation reduces to a simple shift in registers for some specific divisors.

As explained in Section 6.2, AC-CCDM can be realized by splitting an interval into $n_{a}$ per 1-D. This requires at most $n_{a}$ multiplications. For each multiplication, one of the multipliers is found by a division using the statistics of the composition. Finally, at most $n_{a}$ comparisons are carried out. We note that practical discussions such as “numerical precision”, “gaps between intervals” and “rescaling” are omitted here, and the reader is referred to [56,106,107] for details.

An approximate implementation of AC-CCDM is proposed in [68] where computations are realized with fixed-point operations. However, this implementation also requires multiplications, divisions and comparisons of large integer numbers. In addition, an implementation of AC-DM based on finite-precision arithmetic is provided in [72].

SR-CCDM, in contrast to AC, is based on calculating BCs. Thus, the number of required arithmetic operations depends a lot on how this computation is implemented or whether the BCs can be pre-computed and stored.

When ESS and (Algorithm 1 in [23]) are implemented with FP, at most $n_{a}$ additions (subtractions) of numbers from the corresponding shaping matrix are required per 1-D. These numbers are at most $\left\lceil nR_{s}\right\rceil$-bit long. Thus, the computational complexity of these algorithms is O(n). FP implementation of SM however, requires at most *L* multiplications of numbers from the shaping matrix. Therefore, the computational complexity of SM is $O(n^{3})$.

**Example** **12** (FP SpSh, computational complexity). *Based on Example 6, at most four 113-bit additions per 1-D are necessary to realize ESS and (Algorithm 1 in [23]). On the contrary, for SM algorithms, at most 89 113-bit multiplications per 1-D are required.*

With BP approach, ESS and (Algorithm 1 in [23]) can be implemented with at most $n_{a}$ additions of $n_{m}$-bit numbers per 1-D. Then their computational complexity is O(logn). On the other side, BP SM can be realized with at most *L* multiplications of $n_{m}$-bit numbers per 1-D. Therefore, the complexity of SM is now $O(n\log^{2}n)$.

**Example** **13** (BP SpSh, computational complexity)**.** *When Example 6 is now constructed with $n_{m}=9$ and $n_{p}=7$, ESS and (Algorithm 1 in [23]) require at most four 9-bit additions per 1-D. Correspondingly, if SM is realized with $n_{m}=6$ and $n_{p}=7$, at most 89 6-bit multiplications are necessary per 1-D.*

Table 3 summarizes serialism, required storage and computational complexity of discussed shaping algorithms as classified in Figure 1. The main conclusion from Table 3 is that for DM, AC and SR provide a tradeoff between serialism and computational complexity. However, we note that SR can only be used for binary-output DM. On the other hand for SpSh, SM and ESS create a tradoff between required storage and computational complexity. The selection among different algorithms then depends on the actual resources that are available for shaping in practice, and thus, we refrain from making definitive suggestions here.

**Figure 12:**
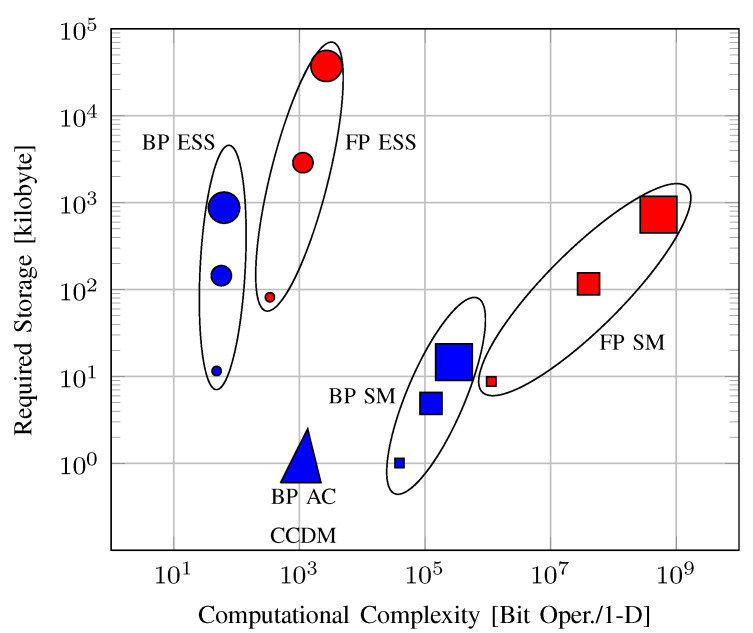
Approximate illustration of maximum computational complexity vs. maximum required storage for ESS, SM and CCDM. Red- and blue-colored markers indicate FP and BP implementations, respectively. Radii of the markers are proportional to the corresponding blocklength n∈{64,216,512}. Here we assume that BP AC-CCDM is implemented with finite-precision arithmetic using 16-bit numbers which is comparable to the values selected in [72]. Furthermore, we assume that a *k*-bit addition is equivalent to *k* bit operations, while a *k*-bit multiplication is equivalent to $k^{2}$ bit operations as in [23].

We conclude this paper by showing in Figure 12, the maximum required storage versus maximum number of computations required to implement BP and FP SpSh, and BP AC-CCDM (BP AC-CCDM refers to the finite-precision implementation of AC-CCDM as discussed in [72]). We see that there is a computational complexity vs. required storage tradeoff between ESS (and (Algorithm 1 in [23])) and SM. ESS requires larger storage but can be implemented with a smaller complexity, and only demands additions and subtractions. On the other hand, SM can be realized with a smaller storage, however requires many multiplications and divisions. In fact, by modifying the corresponding shaping and deshaping algorithms, it is also possible to adjust the balance between computational complexity and required storage as explained in (Section 4.3.4 in [10]), i.e., operate between the ESS and SM clusters in Figure 12. Furthermore, there is also a difference in computational complexities of ESS and (Algorithm 1 in [23]). An initial step is required in (Algorithm 1 in [23]) where the *n*-shell that the corresponding sequence is located on is determined. This step requires at most L−1 additions and comparisons.

Finally, Figure 12 also shows that BP AC-CCDM can be implemented with moderate computational complexity and minimal storage. Furthermore, these requirements do not heavily depend on blocklength *n*. Therefore, for large *n* where its rate losses are small, and for applications for which high serialism of AC is not problematic, AC-CCDM is an effective and low-complexity choice as a shaping algorithm.

## 7. Conclusions

This paper reviewed prominent amplitude shaping architectures and algorithms for the probabilistic amplitude shaping (PAS) framework. Constant composition distribution matching (CCDM), multiset-partition DM (MPDM) and sphere shaping (SpSh) are all optimum shaping techniques for asymptotically large blocklengths, in the sense that they have vanishing rate loss. However, for short blocklengths, CCDM addresses a smaller set of output sequences than that of MPDM and SpSh, leading to higher rate losses. We provided evidence for the AWGN channel that seeking to utilize the signal space in energy-efficient manners is better than attempting to obtain the capacity-achieving distribution, which is derived for asymptotically large, and thus, impractical blocklengths. Therefore, MPDM, SpSh, and other energy-efficient shaping architectures are suitable to be used over a wider blocklength regime, especially for blocklengths below a couple of hundred symbols.

In addition to the rate loss analysis, we evaluated information rates and frame error rates (FER) of PAS employing CCDM, MPDM and SpSh as the amplitude shaping architecture. Enumerative sphere shaping (ESS) and shell mapping (SM) are both considered as potential SpSh algorithms. AWGN channel simulations with 64-QAM demonstrate that power-efficiency gains on the order of 1 dB can be obtained already at blocklengths around 200 by employing MPDM and SpSh, and thus, justify our earlier observation on the objective of amplitude shaping. CCDM provides gains around 0.75 dB for the same settings. Furthermore, these gains are predicted well by shaping gain and information rate computations based on bit-metric decoding.

In the last part of the paper, we discussed the performance of shaping algorithms considering latency, required storage and computational complexity. To realize DM, arithmetic coding (AC)-based implementation of MPDM requires minimal storage and can be implemented with a few computations per input symbol. However, AC has a higher serialism than subset ranking (SR)-based implementation which on the other hand has increased computational complexity. For SpSh, ESS and SM provide a tradeoff between storage and computational complexities, where the complexity is more due to the required storage for ESS and required number of computations for SM. Thus, the decision on which algorithm should be used to realize energy-efficient amplitude shaping depends on the application-specific requirements on latency, available storage and tolerable computational complexity.

## Figures and Tables

**Table 1:** Content of an amplitude sequence as in Figure 4 based on Example 2.

Parameter	Formula (per *n*-Sequence)	Value per 1-D (Example 2)	Value per 216-D (Example 2)
Data on amp.	nH(A)	1.75	378
Data on sign	nγ	0.50	108
Shap. redundancy	n(m−1−H(A))	0.25	54
Cod. redundancy	n(H(A)+1−R)	0.50	108
Redundancy	n(m−R)	0.75	162
Data, nR	n(H(A)+γ)	2.25	486

**Table 2:** Parameters Computed in Example 8.

Architecture	*k*	k/n	*E*	$H(\bar{A})$ or $H(\tilde{A})$	*R*_loss_
CCDM	367	1.6991	11.00	1.7504	0.0513
MPDM	374	1.7315	11.00	1.7504	0.0189
SpSh	374	1.7315	10.90	1.7448	0.0133

**Table 3:** Serialism, required storage and computational complexity.

	Direct Method (Distribution Matching)		Indirect Method (Energy-Efficient Signal Space)	
	AC-CCDM [52]	SR-DM [58]	ESS [22] and (Algorithm 1 in [23])	SM [23]
Serialism (no. of loop iter.)	k+n	$\min(n_{1},n-n_{1})+1$	k+n	$k+\log_{2}n$
Storage Complexity	O(logn)	O(logn)	FP: $O(n^{3})$ BP [65]: $O(n^{2}\log n)$	FP: $O(n^{2}\log n)$ BP [65]: $O(n\log^{2}n)$	
Computations (per 1-D)	$n_{a}$ divisions, multiplications and comparisons	Sh: $\left(n_{a}-1\right)$ BCs Dsh: $(n_{a}-1)/2$ BCs	Sh: $n_{a}$ comparisons and subtractions Dsh: $n_{a}$ additions (and *L* comparisons/additions per *n*-D for [23, Algorithm 1])	Sh: *L* multiplications, comparisons and subtractions ${}^{†}$ Dsh: *L* multiplications and additions

${}^{†}$ SM requires a division per dimension for shaping as well. (Sh:Shaping, Dsh: Deshaping, BC: Binomial Coefficient).

## Abbreviations

The following abbreviations are used in this manuscript:

AC	Arithmetic coding
AIR	Achievable information rate
ASK	Amplitude-shift keying
AWGN	Additive white Gaussian noise
BC	Binomial coefficient
BL-DM	Bit-level distribution matching
BMD	Bit-metric decoding
BP	Bounded-precision
BRGC	Binary reflected Gray code
CCDM	Constant composition distribution matching
CM	Coded modulation
D&C	Divide and conquer
DEMUX	Demultiplexer
DM	Distribution matching
ESS	Enumerative sphere shaping
FEC	Forward error correction
FER	Frame error rate
FP	Full-precision
GMI	Generalized mutual information
GS	Geometric shaping
LDPC	Low-density parity-check
LLR	Log-likelihood ratio
LUT	Lookup table
MB	Maxwell-Boltzmann
MC	Multinomial coefficient
MI	Mutual information
MLC	Multilevel coding
MPDM	Multiset-partition distribution matching
MUX	Multiplexer
PA	Parallel-amplitude
PAS	Probabilistic amplitude shaping
PDM	Product distribution matching
PMF	Probability mass function
PS	Probabilistic shaping
QAM	Quadrature amplitude modulation
SM	Shell mapping
SNR	Signal-to-noise ratio
SpSh	Sphere shaping
SR	Subset ranking
